# Lower help-seeking intentions mediate subsequent depressive symptoms among adolescents with high autistic traits: a population-based cohort study

**DOI:** 10.1007/s00787-021-01895-3

**Published:** 2021-10-25

**Authors:** Mariko Hosozawa, Syudo Yamasaki, Shuntaro Ando, Kaori Endo, Yuko Morimoto, Sho Kanata, Shinya Fujikawa, Noriko Cable, Hiroyasu Iso, Mariko Hiraiwa-Hasegawa, Kiyoto Kasai, Atsushi Nishida

**Affiliations:** 1 Institute for Global Health Policy Research, Bureau of International Health Cooperation, National Center for Global Health and Medicine, 1-21-1 Toyama, Shinjuku-ku, Tokyo, 162-8655 Japan; 2 Department of Epidemiology and Public Health, University College London, London, UK; 3 Department of Pediatrics and Adolescent Medicine, Juntendo University, Tokyo, Japan; 4 Research Center for Social Science & Medicine, Tokyo Metropolitan Institute of Medical Science, Tokyo, Japan; 5 Department of Neuropsychiatry, Graduate School of Medicine, The University of Tokyo, Tokyo, Japan; 6 Department of Psychology, Ube Frontier University, Ube, Japan; 7 Department of Psychiatry, Teikyo University School of Medicine, Tokyo, Japan; 8 Department of Social Medicine, Public Health, Osaka University Graduate School of Medicine, Osaka, Japan; 9 Department of Evolutionary Studies of Biosystems, The Graduate University for the Advanced Studies, SOKENDAI, Hayama, Japan

**Keywords:** Autistic traits, Depression, Help-seeking, Adolescence, Population-based study

## Abstract

Adolescents with high autistic traits are at increased risk of depression. Despite the importance of seeking help for early intervention, evidence on help-seeking intentions amongst this population is scarce. Using a population-based cohort in Japan, we examined adolescents’ help-seeking intentions and preferences by the level of autistic traits and tested its mediating role on the association between high autistic traits and depressive symptoms. At age 12, we measured parent-rated autistic traits using the short version of the Autism Spectrum Quotient and classified the adolescents into two groups (≥ 6 as AQhigh, < 6 as AQlow); help-seeking intentions and preferences were assessed through a depression vignette. At age 14, depressive symptoms were self-rated using the Short Mood and Feelings Questionnaire. Hypothesised associations between autistic traits and help-seeking intentions or depressive symptoms were tested applying multivariable regression modelling, while mediation was tested with structural equation modelling. Of the 2505 adolescent participants, 200 (8%) were classified as AQhigh. In both groups, the main source of help-seeking was their family; however, 40% of the AQhigh group reported having no help-seeking intentions compared to 27% in the AQlow. The AQhigh group was at increased risk of not having help-seeking intentions (OR 1.84, 95% CI 1.35–2.50) and higher depressive symptoms (b coefficient 1.06, 0.33–1.79). Help-seeking intentions mediated 18% of the association mentioned above. Interventions to promote help-seeking intentions among adolescents with high autistic traits could reduce their subsequent depressive symptoms. Ideally, such interventions should be provided prior to adolescence and with the involvement of their parents.

**Supplementary Information:**

The online version contains supplementary material available at 10.1007/s00787-021-01895-3.

## Introduction

Autism spectrum disorder (hereafter referred to as autism) is a neurodevelopmental condition that is characterised by difficulties in social communication and interaction and restricted, repetitive patterns of behaviour, interests, or activities [1]. Autism is a dimensional condition, and autistic traits exist on a continuum across the general population [2]. Those diagnosed with autism and those undiagnosed with high autistic traits are at increased risk of mental health problems. For example, around 70–80% of all people with autism experienced mental health problems, such as depression [3], which may increase during adolescence [4]. Studies targeting adolescents with high autistic traits in the general population showed approximately a twofold increased risk of depression and suicidal behaviour among them [5, 6].

Help-seeking intentions facilitate early intervention, which is essential for preventing or reducing mental health problems [7]. However, qualitative studies from European countries showed that even in countries with good access to health care, autistic individuals reported reluctance in seeking help for their mental health problems, partly due to their difficulties in recognising the need for help and stronger inclinations for self-reliance [8, 9]. In general, the tendency for reluctance to seek help may be more prevalent in the culture of Asian origin, including Japan, as people may be more reluctant to ask for support from others [10]. Furthermore, help-seeking intentions for mental health problems may be lowest in early adolescence [11], while many mental health problems first emerge during this period [7].

Overall, existing evidence on the mental health of adolescents with high autistic traits underscores the need for approaches to prevent mental health problems, regardless of whether a formal diagnosis is given. Interventions to promote help-seeking intentions during early adolescence could be effective. However, there is limited evidence on help-seeking intentions for mental health problems among adolescents with high autistic traits, and evidence that examined the effect of help-seeking intentions on subsequent mental health problems in this population is lacking. Establishing evidence in this area will deepen our understanding of the role of help-seeking intentions in preventing subsequent depressive symptoms among individuals with high levels of autistic traits, both diagnosed and undiagnosed.

Using the Tokyo Teen Cohort (TTC) study, an ongoing population-based cohort study of adolescents living in Tokyo, our primary aim was to assess help-seeking intentions related to depression and help-seeking preferences according to the level of autistic traits with a focus on early adolescence. We also aimed to examine the potential mediating role of help-seeking intentions in the pathway from autistic traits to subsequent depressive symptoms during adolescence. We hypothesised that adolescents with high autistic traits would show decreased help-seeking intentions, which would explain their increased risk of subsequent depressive symptoms.

## Methods

### Study population

The TTC study is an ongoing population-based cohort study, following the physiological and psychological development of 3171 children born between 2002 and 2004 and living in three municipalities in the metropolitan area of Tokyo, Japan, from 10 years of age. Detailed information about the TTC study is described elsewhere [12]. Of the 3,007 children who took part in the TTC study at age 12 (when information on their autistic traits and help-seeking intentions was collected), those without valid information on their help-seeking intentions or autistic traits (*n* = 482 and 20, respectively) were excluded, leaving 2505 adolescent participants in our study. All procedures involving human participants were approved by the institutional review boards of the Tokyo Metropolitan Institute of Medical Science (approval number: 12–35), SOKENDAI (Graduate University for Advanced Studies, 2,012,002), and the University of Tokyo (10,057). Written informed consent was obtained from all the parents of the participating children, and informed assent was obtained from the children. The reporting of this study follows the Strengthening the Reporting of Observational Studies in Epidemiology (STROBE) guidelines.

### Age 12 autistic traits

The participants’ autistic traits were measured at age 12 (wave 2) using the short form of the Autism Spectrum Quotient adolescent version, a parent-rated questionnaire designed to measure autistic traits in the general population [13, 14]. The questionnaire consisted of 10 items that were given a score of either 0 or 1. We calculated the total score (range 0–10, where higher total scores indicated greater autistic tendencies) and classified the participants that scored above six as high autistic traits (AQhigh) as opposed to those who were not (AQlow), guided by findings from the previous study [14].

### Age 12 help-seeking intentions for depression

We measured the participants’ help-seeking intentions for depression at age 12 using a vignette written in Japanese. The vignette presented a boy named ‘Taro’ (a popular boys’ name in Japan) who was meant to display symptoms of depression. The symptoms presented in the vignette covered the Diagnostic and Statistical Manual of Mental Disorders (Fourth Edition) and International Classification Of Diseases 10 criteria for major depression and mimicked those used in other large-scale studies [15]. Following is an example of the vignette: ‘For the last several weeks, Taro has been feeling unusually sad. He is tired all the time and has trouble sleeping at night. Taro does not feel like eating and has lost weight. He cannot keep his mind focused on his studies, and his grades have dropped. He puts off making any decisions and even day-to-day tasks, such as studying and extracurricular activities, seem too much for him. His parents and teachers are very concerned about him’. Following the vignette, participants were asked four questions regarding their help-seeking intentions and preferences, which are described below.

1. *Recognition of the need for help in the vignette case* Adolescents were asked, ‘What do you think about Taro's condition?’ The response options were (1) ‘He needs help’; (2) ‘He has a problem but does not need help’; or (3) ‘He does not have a problem’.
2. *Help-seeking intentions for depression* Adolescents were asked to respond to the question, ‘If you were in the same situation as Taro, would you seek help from others?’ The response options were (1) ‘I would consult someone immediately’ or (2) ‘I would wait and see without consulting anyone’, from which we created a binary variable based on their response.
3. *The number of people whom the adolescent could rely on for help (I have someone to rely on for help)* Adolescents were asked, ‘If you were in the same situation as Taro, and if you considered asking someone for help, how many people do you think you could rely on?’ The participants were given the following options: Between 0 and 4 people and 5 or more people. The responses were summarised into a binary variable that represented having no one vs having someone, given that not having anyone to rely on for help had a particular impact on mental health [16].
4. *Assessment of help-seeking preferences* Adolescents were asked, ‘If you were in the same situation as Taro, who would you ask for help?’ The participants had to select as many options as were relevant from the following list: ‘Friends’, ‘Family’, ‘Relatives’, ‘Teachers’, ‘School counsellor’, ‘Medical doctor’, or ‘Online forum’. All responses were treated in a binary format (not selected vs selected).

### Age 14 depressive symptoms

The participants’ depressive symptoms were measured using the Short Mood and Feelings Questionnaire (SMFQ) administered at age 14 [17]. The SMFQ is a well-validated 13 item self-reported questionnaire for children and adolescents to evaluate their depressive symptoms in the preceding 2 weeks (range 0–26, where higher scores indicated more severe depressive conditions) [18]. The original version of the SMFQ has been used in several studies on adolescents with autism [5]. The Japanese version has been used in other population-based studies in Japan [19], and Cronbach's alpha for this scale was 0.91 in our study.

### Covariates

Based on previous studies, the following six covariates, all measured at age 10, were included in our model as potential confounders [16]: the child's sex; absolute poverty of the household (as indicated by the report of an annual household income below 4,000,000 Yen, which is just below the median national income in Japan); mother’s highest level of education (graduated from high school or higher); child's intelligence quotient (assessed through an interview that used the short-form of the Wechsler Intelligence Scale for Children) [20]; parent-rated emotional symptoms measured using the emotional difficulties subscale of the Strengths and Difficulties Questionnaire (SDQ, range 0–10, where higher scores indicated more severe emotional problems) [21]; and parental help-seeking intentions which were measured using the adult-version of the depression vignette. The adult-version of the vignette, which mimicked that used in a previous study [22], largely replicated the child-version but described a 30-year-old named Suzuki (a common surname in Japan) instead. In our sample, the prevalence of adolescents with both parents being non-Japanese was low (< 0.1%); therefore, we did not include this factor in our study model.

### Statistical analyses

We first conducted a descriptive analysis to examine the association between autistic traits and our study variables. We then applied multivariable logistic and regression analyses to examine the association between (1) autistic traits and help-seeking intentions (logistic) and (2) autistic traits and self-rated depressive symptoms at age 14 (linear regression). In both analyses, an unadjusted model (Model 1), a model that was adjusted for sex (Model 2), and a model that was further adjusted for potential confounders (i.e., low annual household income, low maternal education, parental help-seeking intentions, child's intelligence quotient, and parent-rated emotional symptoms [Model 3]) were examined. Our preliminary analyses showed no evidence suggesting differences in associations according to sex or level of parent-rated emotional symptoms at age 10. Possible mediation by the child's help-seeking intentions on the association between autistic traits and depressive symptoms at age 14 was tested in terms of the direct and indirect effects.

We conducted several sensitivity analyses. A sample bias analysis was implemented to examine differences between our analytic sample and those excluded from our study in relation to our study variables. We repeated the analyses by excluding adolescents whose parents reported receiving a diagnosis of autism by age 12 (*n* = 30), because receiving a formal diagnosis could influence the child’s help-seeking intentions and subsequent depressive symptoms. Furthermore, we repeated the mediation analysis by replacing self-reported depressive symptoms with parent-rated emotional symptoms measured using the emotional difficulties subscale of SDQ measured at age 14. This was undertaken to examine whether the mediating role of the child’s help-seeking intentions would be consistent across two different sources of informants. Descriptive analyses were conducted using Stata SE version 16.1 (StataCorp, College Station, TX, United States). All other analyses were performed using structural equational modelling in Mplus8.5 (Muthén & Muthén, Los Angeles, CA, United States).

### Missing data

The missing data from each variable ranged from 0.04% (on the child’s intelligence quotient) to 27% (on the depressive symptoms at age 14). We imputed missing covariates and outcomes using multiple imputation by chained equations to minimise data loss [23]. Regression analyses were run across 27 imputed data sets and adjusted using Rubin's rules. The imputed results were broadly similar to those obtained using the observed cases (Tables S1 and S4); therefore, the former is presented here.

### Patient and public Involvement

The participants’ advisory board of the TTC study, which consisted of the main carers for the adolescents, provided feedback on the research questions, outcome measures, and design or implementation of the study. None of the board members were asked to advise on the interpretation or writing of the results; however, they were disseminated to the study participants through their dedicated website: http://ttcp.umin.jp/.

## Results

Of the 2505 adolescents in our study sample, 200 (8.0%) were classified into the AQhigh group (Table 1). Compared to the adolescents in the AQlow group, those in the AQhigh group were more likely to be boys (68.0% vs 51.6%, *p* < 0.001) or had received a diagnosis of autism (7.6% vs 0.7%, *p* < 0.001, Table S1). However, the two groups did not differ in terms of the family’s socioeconomic status or children’s mean intelligence quotient (Table 1). Moreover, parents of the adolescents in the AQhigh group were less likely to have help-seeking intentions (71.0% vs 79.5%, *p* = 0.005). The results of our sample bias analysis showed that adolescents whose parents reported a diagnosis of autism by age 12 were more likely to be excluded from our study (Table S2).

**Table 1:** Descriptive characteristics for the imputed sample (*N* = 2505)

	AQlow^a^ (92.0%)	AQhigh^a^ (8.0%)	*p* value^b^
Autistic traits at age 12, mean (SE)	2.1 (0.0)	6.7 (0.1)	< 0.001
Sex			
Male	51.6	68.0	< 0.001
Female	48.4	32.0	
Low annual household income^c^			
No	89.6	88.1	0.53
Yes	10.4	11.9	
Low maternal education^d^			
No	84.0	81.3	0.33
Yes	16.0	18.7	
Parental help-seeking intentions^e^			
No	20.5	29.0	0.005
Yes	79.5	71.0	
Child intelligence quotient, mean (SE)	108.1 (0.3)	106.8 (1.1)	0.24
Parent-rated emotional symptoms, mean (SE)	1.5 (0.0)	2.5 (0.2)	< 0.001
Self-reported depressive symptoms at age 14, mean (SE)	3.0 (0.1)	4.1 (0.4)	0.007

Variables were measured in the first wave of the study (age 10) unless otherwise stated

*AQ* autism spectrum quotient, *SE* standard error

^a^Defined as scoring above the suggested clinical–cutoff value on the short version of the Autism Spectrum Quotient adolescent version as measured by a parent at age 12

^b^*p* value for group difference obtained from the *F* statistic tests

^c^Defined as a household income below 4,000,000 Yen

^d^Defined as a high school graduate or below

^e^Measured through the adult version of the depression vignette

### Help-seeking intentions and preferences by level of autistic traits

**Fig. 1:**
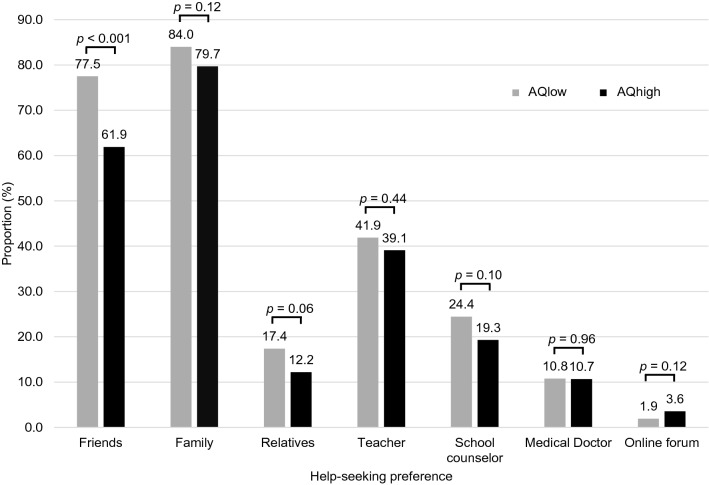
Help-seeking preferences for depression according to the level of autistic traits. The proportion of adolescents that reported seeking help from each source according to the autistic traits, based on observed results. Participants were able to select multiple responses. The *p* value for the group differences was obtained from Chi-square tests. *AQ* autism spectrum quotient

Significantly higher proportion of adolescents in the AQhigh group reported not having help-seeking intentions at age 12 as compared to those in the AQlow group (40.0% vs 26.5%, *p* < 0.001, Table 2). However, both the AQhigh and AQlow groups responded similarly regarding the recognition of the need for help in the vignette (92.5% vs 94.4%, *p* = 0.50) and having someone to ask for help (96.5% vs 98.1%, *p* = 0.11). As shown in Fig. 1, fewer adolescents in the AQhigh group reported seeking help from friends than the AQlow group (61.9% vs 77.5%, *p* < 0.001); however, adolescents from both groups were most likely to seek help from their families (79.7% in AQhigh vs 84.0% in AQlow), followed by their friends. They were less likely to seek help from professionals, such as school counsellors (19.3% vs 24.4%, *p* = 0.10) or medical doctors (10.7% vs 10.8%, *p* = 0.96).

**Table 2:** Adolescents' help-seeking intentions for depression according to the level of autistic traits (*N* = 2505)

	AQlow (*n* = 2305, 92.0%)		AQhigh (*n* = 200, 8.0%)		*p* value^a^
	*n*	%	*n*	%	
Did not have help-seeking intentions	611	26.5	80	40.0	< 0.001
Recognition of the need for help in the vignette					
He needs help	2176	94.4	185	92.5	0.50
He has a problem but does not need help	100	4.3	11	5.5	
He does not have a problem	29	1.3	4	2.0	
[I have] someone to rely on for help	2268	98.1	192	96.5	0.11

All variables were measured through the depression vignette at age 12

*AQ* autism spectrum quotient

^a^*p* value for the group difference was obtained from the Chi-square tests

Our multivariable logistic analysis confirmed the relationship between autistic traits and not having help-seeking intentions (Table 3). After adjusting for covariates, the AQhigh group showed 1.84 times greater odds (95% CI 1.35–2.50, [Model 3]) for not having help-seeking intention than the AQlow group.

**Table 3:** Adolescents’ help-seeking intentions according to the level of autistic traits (N = 2,505)

Adolescent that did not have help-seeking intentions	Model 1 Crude		Model 2 Sex adjusted		Model 3 Covariate adjusted	
	OR	95% CI	OR	95% CI	OR	95% CI
AQhigh (ref = AQlow)	1.85***	1.37–2.49	1.81***	1.34–2.44	1.84***	1.35–2.50
Sex (ref = female)			1.14	0.96–1.37	1.11	0.93–1.33
Low annual household income					1.03	0.77–1.39
Low maternal education					1.11	0.87–1.42
Parental help-seeking intentions					0.74**	0.60–0.92
Child intelligence quotient					1.01**	1.00–1.02
Parent-rated emotional symptoms					0.98	0.92–1.03

*CI* confidence intervals, *OR* odds ratio, *AQ* autism spectrum quotient, *ref* reference

*p < 0 .05, **p < 0.01, ***p < 0.001

### Depressive symptoms by level of autistic traits and the mediating role of help-seeking intentions

At age 14, adolescents in the AQhigh group showed significantly higher mean self-rated depressive symptoms than those in the AQlow group (4.1 vs 3.0, *p* = 0.007, Table 1). In our multivariable regression analysis, being in the AQhigh group was associated with significantly higher depressive symptoms (*b* = 1.06, 0.33–1.79 for Model 3, Table S3). Mediation analysis revealed that not having help-seeking intentions at age 12 partially mediated the association between AQhigh and depressive symptoms at age 14 (approximately 18%, Table 4). Our sensitivity analysis, excluding children diagnosed with autism by age 12 or replacing self-rated depressive symptoms with parent-rated emotional symptoms, showed similar results (Tables S5–S7).

**Table 4:** Estimates of direct and indirect effect (mediated through adolescent’s help-seeking intention) in the association between autistic traits and depressive symptoms at age 14

	Indirect effect	Direct effect	Total effect
Depressive symptoms	*b* (95% CI)	*b* (95% CI)	*b* (95% CI)
AQlow	Ref	Ref	Ref
AQhigh	0.19 (0.06–0.32)**	0.87 (0.22–1.52)**	1.06 (0.41–1.72)**

Mediating effect examined using structural equation modelling in Mplus adjusted for sex, low annual household income, low maternal education, parental help-seeking intention, the adolescent’s intelligence quotient and parent-rated emotional symptoms are shown

*AQ* autism spectrum quotient

*p < 0.05, **p < 0.01

## Discussion

Using a large population-based cohort, we systematically examined the role of help-seeking intentions on the association between high autistic traits and subsequent depressive symptoms. We found that in early adolescence (i.e., age 12), adolescents with high autistic traits were at an approximately 1.8 times higher risk of not having help-seeking intentions for depression than those in the low range of autistic traits. This lack of help-seeking intentions mediated approximately 18% of the association between high autistic traits and subsequent depressive symptoms at age 14. Moreover, although fewer adolescents in the AQhigh group reported friends as their help-seeking source, the main source of help-seeking was their family, regardless of the level of their autistic traits.

Our results support our hypothesis that adolescents with high autistic traits would show less help-seeking intentions, partially explaining their increased risk for subsequent depressive symptoms. To our knowledge, this is the first study to assess help-seeking intentions and preferences of adolescents with high autistic traits in the general population. Our findings were consistent with self-reports of difficulty or reluctance towards seeking help for mental health problems among adults with autism reported in previous qualitative studies [8, 9]. We confirmed that, in the community setting, this lack of help-seeking intention was already present in early adolescence (i.e., by age 12) among adolescents with high autistic traits.

The lack of help-seeking intentions among adolescents with high autistic traits was not a result of their inability to recognise the problem, as most (93%) of them were able to identify the need for mental health support in the vignette case. It was also not due to the lack of a source on which they could rely for help, as adolescents from both groups reported similar, adequate accessibility to someone they could rely on (97% and 98%, respectively). Previous studies reported that autistic adults had difficulties expressing their emotional distress to others, negative views on help-seeking based on their past experiences, and concerns over the stigma attached to mental illness [8, 9]. Investigating these possible mechanisms was beyond the scope of our study; however, these factors could have played a role in forming attitudes toward help-seeking among adolescents with high autistic traits. Future studies clarifying the pathway to decreased help-seeking intentions among adolescents with high autistic traits will offer additional implications for tailored interventions to promote help-seeking in this population.

In our study, help-seeking intentions mediated approximately 18% of the association between high autistic traits and depressive symptoms at age 14. Despite many studies focusing on help-seeking as a target for preventive interventions, longitudinal evidence for the association between help-seeking intentions and subsequent mental health among children is limited [24–26]. Although the effect is modest, our results suggest that promoting help-seeking intentions among children with high autistic traits may help reduce their risk of developing depressive symptoms during adolescence. This is inferred from a recent pilot randomised control trial of a school-based mental health literacy program that reported the effectiveness of their modified programs in meeting the needs of students with developmental disorders, including autism [27]. Given that individuals with high autistic traits already express fewer help-seeking intentions in early adolescence, ideally, the relevant interventions should be provided from before the children enter adolescence by modifying the interventions to ensure age-appropriate content.

As reported in the general population [28], we found that the main source of help sought by adolescents with high autistic traits was their family. Previous studies have shown the importance of families as help-seeking partners throughout childhood and adolescence [28]. For adolescents with high autistic traits, the role of the family as help-seeking partners may be more important than those in the lower range, as they reported significantly less help-seeking towards their friends, another main source of a help-seeking partner during adolescence.

Importantly, in our study, parents of the adolescents with high autistic traits also showed significantly less help-seeking intentions than parents of those in the lower range group. This may correspond with the results of a recent systematic review that reported more avoidance and less social support-seeking as coping strategies among parents of children with autism compared to parents of typically developing children [29]. Given that parental help-seeking intentions had a significant effect on the adolescents’ help-seeking intentions and that parents are often the gatekeepers to formal support for their children [30], offering parents of adolescents with high autistic traits information regarding when and where to ask for mental health support and empowering them to seek help may be beneficial for the child.

### Strengths and limitations

Our study had many strengths, including the use of a population-based cohort that allowed us to compare the results according to the level of autistic traits, and the prospective longitudinal design allowed us to establish the direction of associations. We used self-reports from the adolescents to assess their help-seeking intentions and depressive symptoms. Our sensitivity analysis using parent-rated emotional symptoms further strengthened our result by replicating the observed association across different informants. In addition, the participant’s autistic traits were measured by their parent, which reduced the possibility of shared method variance.

Our study also had several limitations. First, although there were no differences in the proportion of children’s sex in our models, this may have been due to the small number of girls with high autistic traits included in our study. However, the proportion of girls with high autistic traits in our study was similar to those found in other population-based studies [5]. Second, over 90% of the adolescents with high autistic traits had intellectual abilities within the typical range (results provided upon request), indicating that our results are less likely to apply to autistic adolescents with cognitive delay. Nevertheless, autistic individuals with no cognitive delay are more prone to mental health problems [31] and are more likely to remain undiagnosed than their counterparts [32]. Given that, our findings are relevant to autistic adolescents who are community bounded. Third, we relied on self or parent-reported questionnaires to gather information, including autistic traits and depressive symptoms. Using objective measures or structured interviews in the future will further validate the observed association. Forth, adolescents with high autistic traits may have had difficulties interpreting the vignette or articulating their depressive symptoms [33]. However, almost all the adolescents from both groups recognised the presence of mental health problems and the need for help in the vignette. Moreover, the SMFQ has reliably been used among autistic adolescents in previous studies [5]. Relatedly, using a vignette to measure help-seeking intentions likely helped our participants conceive what they would do if they had similar experiences as in the vignette (i.e., depressive symptoms) [34]. The vignette in our study featured a scenario of a boy. Although no further information is available to us, future studies could include both a boy’s and a girl’s name in the vignette to reduce the possibility of response bias due to the perceived gender of the name used [25]. In addition, including standardised composite measures of help-seeking intentions, such as the General Help-Seeking Questionnaire [35], will be needed to measure the degree of help-seeking intentionality and aid comparison across studies. Fifth, we did not have information on the actual help-seeking behaviours (i.e., actual service use) of the adolescents at age 14, which could have influenced the adolescents’ depressive symptoms. However, this is likely to lie within our hypothesised pathway from help-seeking intentions to subsequent depressive symptoms, as the theory of planned behaviour asserts that help-seeking intentions are a determinant of behaviour, and help-seeking intentions have been shown to correlate with actual service use [36]. Finally, cultural and country-specific contexts could influence the notion of seeking help for mental health problems in our study setting. Some studies implied increased hesitancy to seek help in Asian cultures [10]. However, the overall proportion of help-seeking intentions in our study was relatively similar to what was reported in a recent review, mainly from western countries [25], suggesting that our findings are likely to be relevant to countries of non-Asian culture as well. Nevertheless, future research needs to explore if the findings of this study could be replicated in countries with different cultures.

## Clinical implications and conclusion

At the cusp of adolescence, children with high autistic traits are already at an increased risk of not having help-seeking intentions for their mental health problems, despite being at greater risk of experiencing mental health difficulties than their counterparts, which healthcare and educational practitioners do should be aware. Many adolescents in our study identified their families as the main source of help. However, the parents of adolescents with high autistic traits also exhibited lower help-seeking intentions, indicating the need to empower and support those parents as well, because they are often the gatekeeper in the way of their child’s access to proper mental health support. Our findings offer initial evidence that promoting help-seeking intentions among adolescents with high autistic traits, ideally through interventions adapted to meet their needs, could help reduce subsequent mental health problems. Further studies that clarify the underlying mechanism between high autistic traits, help-seeking intentions, and the adolescents’ mental health problems will provide additional evidence for the development of these tailored interventions.

## Supplementary Information

Below is the link to the electronic supplementary material.

Supplementary file1 (DOCX 54 kb)

## Data Availability

The data that support the findings of this study are available on request from the corresponding author (MH). The data are not publicly available due to their containing information that could compromise the privacy of research participants.

## Code availability

The code that supports the findings of this study is available on request from the corresponding author (MH).
